# Distribution and outcomes of paediatric anaesthesia services in Sweden: an epidemiological study

**DOI:** 10.1016/j.bja.2024.07.007

**Published:** 2024-08-01

**Authors:** Björn Bergh-Eklöf, Karl Stattin, Ali-Reza Modiri, Robert Frithiof, Peter Frykholm

**Affiliations:** 1 Department of Surgical Sciences, Section of Anaesthesiology and Intensive Care Medicine, Uppsala University, Uppsala, Sweden; 2 Uppsala Centre for Paediatric Anaesthesia and Intensive Care Research, Uppsala University Hospital, Uppsala, Sweden

**Keywords:** adverse events, anaesthesia, centralisation of surgery, children, complications, infants, mortality

## Abstract

**Background:**

Centralisation of perioperative care for small children to a limited number of specialised paediatric centres has many theoretical advantages, but neither the optimal balance nor the current distribution of paediatric anaesthesia on a national scale are well elucidated. The aim of this study was to describe the distribution, adverse events, and mortality in children undergoing anaesthesia in Sweden.

**Methods:**

In this cohort study, data from all paediatric anaesthesia procedures registered in Sweden during the years 2019–22 were extracted from the Swedish Perioperative Register (SPOR). Data were analysed according to hospital level of care and ASA physical status.

**Results:**

Data from 81 hospitals were analysed. During the 4-yr period, 214,964 procedures were registered. Most procedures in neonates and infants were managed in paediatric (73%) and other university hospitals (21%). Adverse events occurred in 2.71% of cases and severe adverse events in 0.067%. The all-cause 24-h mortality rate was 6.6:10,000 anaesthetics and the all-cause 30-day mortality was 14.7:10,000 anaesthetics. The 30-day all-cause estimated mortality was highest in paediatric hospitals (95% confidence interval [CI] 30–39) compared with other university hospitals (95% CI 6.1–12), county (95% CI 1.9–4.8), district (95% CI 0.07–2.2), and smaller hospitals (95% CI 0.0–22).

**Conclusions:**

Most procedures in neonates and infants were performed in tertiary centres, with county hospitals managing mainly older children, in accordance with national recommendations. Mortality was more common in tertiary centres, reflecting increased comorbidity and centralisation of anaesthesia of neonates and infants.

**Editor's key points**

• Surgery services for children are increasingly specialised. In many countries, there is a volume–outcome debate where the distance to local hospitals is a factor for patients and families.
• This debate must be informed by robust routine data rather than by expert views and public opinion.
• This paper gives a robust description of the nature of surgery for children and its provision at a national level, with outcome data.

In Sweden, as in most European countries, there is a movement to centralise the perioperative care of small children to a limited number of specialised paediatric centres.^1^ This is driven by professional organisations and based on results from international audits such as APRICOT and NECTARINE, which have indicated better outcomes with increased experience of the anaesthesia team.^2^^,^^3^ The Safetots Initiative has proposed that specially trained paediatric anaesthetists should treat children younger than 3 yr with underlying congenital and metabolic diseases and children irrespective of age who undergo major or complex surgery.^4^ The rationale is that complications related to anaesthesia are more common in children than in adults, and the level of experience of the attending anaesthetist is inversely related to the rate of complications.^2^^,^^5^ Furthermore, the United Nations Convention on the Rights of the Child states that children have the right to receive the highest attainable standard of care.^6^ Although the centralisation of perioperative care of neonates and infants is a global trend, there is a paucity of data regarding the ideal and real distribution of paediatric anaesthesia procedures within a country. Furthermore, the rate of complications and mortality associated with paediatric anaesthesia at the different levels of care is not well elucidated. The aim of the present study was to investigate the distribution of paediatric anaesthesia in Sweden, and to determine the rate of complications and mortality using data extracted from a national register.

## Methods

This was a register-based cohort study, using comprehensive data from the Swedish Perioperative Register (SPOR)^7^ extracted on March 10, 2023. The Swedish Ethical Review Authority granted permission, waiving informed consent (June 5, 2022; ref 2020-01909). Data were analysed and reported according to the Strength of Reporting Observational data in Epidemiology (STROBE) guidelines.^8^ Data comprised all procedures involving anaesthesia services in children ≤15 yr of age, performed from January 1, 2019, to December 31, 2022, in Sweden. When applicable, data were reported according to age group definitions: neonates (<2 months), infants (2–12 months), toddlers (1–2 yr), early childhood (3–5 yr), and school children (6–15 yr). Note that the upper limit of the neonatal age group differs slightly from the National Institutes of Health (NIH) definitions by using 2 months instead of 28 days.^9^ The term children was used for all ages ≤15 yr. The outcomes were caseload distribution, perioperative adverse events (AEs), severe adverse events (SAEs), and 24-h and 30-day all-cause mortality; data were analysed according to age, ASA physical status,^10^ and hospital category.

The number of procedures and age distribution involving an anaesthesia service was analysed according to the five levels of care used by SPOR: paediatric (university) hospital (tertiary centre with specialised paediatric services including PICU), university hospital (tertiary centre with mixed adult and paediatric service), county hospital (various adult and paediatric services, adult ICU, intermediate level neonatal intensive care), district hospital (basic emergency service with or without paediatric services; no ICU), and small hospital (one or a few specialties represented; no ICU; no regular paediatric services).

The complications registered in SPOR were solely anaesthesia-related, that is, no surgical AEs were included except for massive haemorrhage with multifactorial causes. In Sweden, perioperative AEs are classified according to the Classification of Procedures in Health Care (Klassifikation av vårdåtgärder [KVÅ] by its Swedish acronym) system, issued by the Swedish Board of Health and Welfare.^11^ Complications of grade 0 designate absence of AEs. True AEs range from grade 1 (no effect on postoperative care), grade 2 (affects provision of care in the PACU, but not further postoperative care), grade 3 (affects the provision of care in the postoperative unit with prolonged care, extra observation, or both), grade 4 (affects the provision of care so that postoperative intensive care is required), to grade 5 (entails probable lasting morbidity or mortality). In the present study, an SAE was defined as a registered perioperative complication of grade 4 or 5.^12^ Postoperative AEs were incompletely available and not analysed owing to the low overall registration rate of 60%. Hospitals that did not report complications were excluded from the analysis of AEs but included in the analysis of distribution and mortality (Supplementary Figure S1).

Mortality was based on the registered all-cause deaths in SPOR, extracting 24-h and 30-day mortality from the recorded date of surgery and corresponding date of death. These data are supplied to SPOR from the National Cause of Death Register, hosted by the Swedish Board of Health and Welfare.

### Statistical analysis

Data regarding number of procedures were expressed as counts (%), AEs as counts and incidence per 10,000 anaesthetics. Mortality data were displayed as number of deaths in absolute numbers and probability of death within 24 h and 30 days, with 95% confidence intervals based on the binomial distribution. R version 4.3 (R Foundation for Statistical Computing, Vienna, Austria) was used for the statistical analyses. There were no missing data regarding caseload or mortality. Two hospitals accounting for 17,034 procedures did not report AE data. The overall AE registration frequency in the remaining 79 hospitals was 88% (range 59–100%); AE data were missing in 19% (*n*=40,648 cases) of the full cohort and in 12% (*n*=23,614) of cases when the two hospitals without AE reporting were excluded. A sensitivity analysis was performed, checking for correlation between SAEs and completeness of reporting for each hospital.

## Results

### Distribution

**Fig 1:**
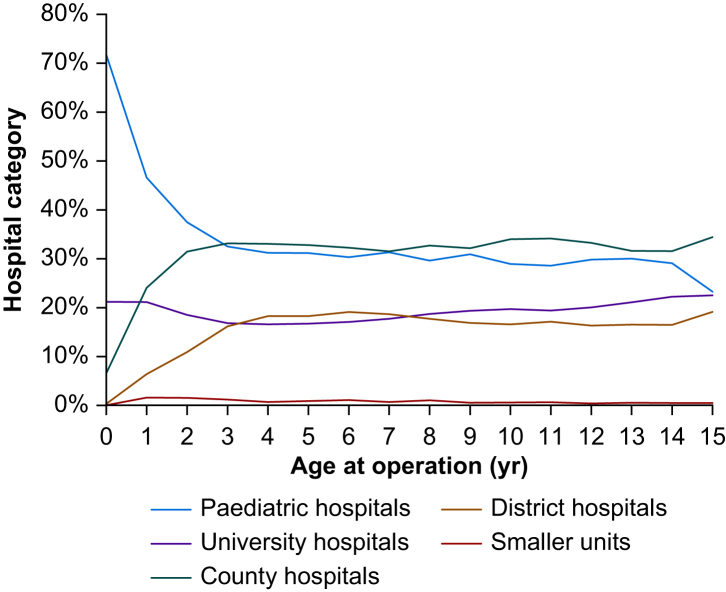
Line graph depicting the proportions of different age groups in the different hospital categories. Neonates and infants were mostly managed in paediatric or other university hospitals.

Data from 81 hospitals were analysed. During the studied 4-yr period, 214,964 procedures were performed on 145,693 children. The age distribution according to hospital category is illustrated in Figure 1 and Supplementary Tables S1 and S2. A total of 4163 (1.9%) procedures were performed in neonates, 12,048 (5.6%) in infants, 14,631 (6.8%) in toddlers, 47,962 (22.3%) in children of early childhood, and 136,160 (63.3%) in school children (Table 1). Furthermore, 97% of neonatal procedures and 92% of procedures in infants were managed in paediatric university hospitals and other university hospitals. Similarly, children aged ≤3 yr were operated in paediatric or university hospitals in 47% and 19% of cases, respectively.

**Table 1:** Number of procedures with anaesthesia categorised according to hospital level and age group of the child. Neonates (<2 months), infants (1–12 months), toddlers (1–2 yr), early childhood (3–5 yr), and school children (6–15 yr). Absolute numbers from the 4 yr of the study (% of row).

Age category	Paediatric hospitals	University hospitals	County hospitals	District hospitals	Smaller units	Grand total
Neonates	3494 (83.9)	553 (13.3)	111 (2.7)	5 (0.1)	0 (0.0)	4163
Infants	8162 (67.7)	2886 (24.0)	956 (7.9)	43 (0.4)	1 (0.0)	12048
Toddlers	12,634 (42.0)	5959 (19.8)	8398 (27.9)	2623 (8.7)	461 (1.5)	30075
Early childhood	15,312 (31.7)	8085 (16.7)	15,969 (33.1)	8493 (17.6)	433 (0.9)	48292
School children	35,221 (29.3)	23,786 (19.8)	39,495 (32.8)	21,110 (17.5)	774 (0.6)	120,386
**Total**	**74,823 (34.8)**	**41,269 (19.2)**	**64,929 (30.2)**	**32,274 (15.0)**	**1669 (0.8)**	**214,964**

The distribution of ASA class is displayed in Table 2 and Supplementary Table S2. Among children with significant comorbidity (ASA >II), 13% were operated in non-paediatric or other university hospital settings.

**Table 2:** All operations with reported ASA classifications, excluding 19,744 operations with no reported or unknown ASA physical status. Hospital categories expressed as absolute number (% of ASA class).

ASA physical status	Paediatric hospitals	University hospitals	County hospitals	District hospitals	Smaller hospitals	Total
1	24,531 (22)	20,118 (18)	42,862 (39)	22,501 (20)	990 (1)	111,002 (57)
2	20,155 (36)	11,945 (21)	16,239 (29)	6642 (12)	604 (1)	55,585 (28)
3	17,179 (64)	6033 (23)	3050 (11)	485 (2)	48 (0)	26,795 (14)
4	1217 (69)	480 (27)	70 (4)	4 (0)	0 (0)	1771 (1)
5	41 (66)	17 (27)	3 (5)	1 (2)	0 (0)	62 (0)
6	1 (20)	3 (60)	0 (0)	1 (20)	0 (0)	5 (0)
**Grand Total**	**63,124 (32)**	**38,596 (20)**	**62,224 (32)**	**29,634 (15)**	**1642 (1)**	**195,220 (100)**

### Adverse events

The incidence of anaesthetics with AEs was 2.6% in paediatric hospitals, 3.6% in university hospitals, 3.5% in county hospitals, 2.4% in district hospitals, and 1.4% in small hospitals. The distribution of AEs according to grade and hospital category is displayed in Supplementary Table S3. The 10 most common AEs are shown in Table 3. Laryngospasm was the most common AE, registered in 725 (0.42%) procedures. The incidence of anaesthetics with SAEs was 7.6:10,000. The 10 most common SAEs are displayed in Table 4. The most common SAE was bronchospasm (22 cases, incidence 1.3:10,000). Twenty-one cases of cardiac arrest were registered, corresponding to an incidence of 1.2:10,000. There were no statistically significant differences of AEs between hospital categories.

**Table 3:** The 10 most commonly registered intraoperative adverse events. Absolute number and incidence (*n*:10,000). The denominator for the incidence calculation was *n*=174,316 (all procedures after exclusion of cases with missing adverse event data).

Name of complication	Count	Incidence
Laryngospasm	726	41.7
Hypothermia	611	35.0
Change of anaesthesia method	530	30.4
Hypoxaemia	500	28.7
Other respiratory complication	473	27.1
Difficult i.v. access	300	17.2
Difficult tracheal intubation	270	15.5
Bradycardia	254	14.6
Bronchospasm	249	14.3
Change of laryngeal mask airway	209	12.0

**Table 4:** The 10 most common severe adverse events. Absolute number and incidence (*n*:10,000). The denominator for the incidence calculation was *n*=174,316 (all procedures after exclusion of cases with missing adverse event data). EBL, estimated blood loss.

Type of complication	Count	Incidence
Bronchospasm	22	1.3
Other respiratory complication	18	1.0
Hypoxaemia	15	0.9
Aspiration	14	0.8
Unplanned reintubation	12	0.7
Laryngospasm	12	0.7
Hypovolaemia	12	0.7
Cardiac arrest	10	0.6
EBL >50% of blood volume	9	0.5
Hypotension	8	0.4

### Mortality

**Fig 2:**
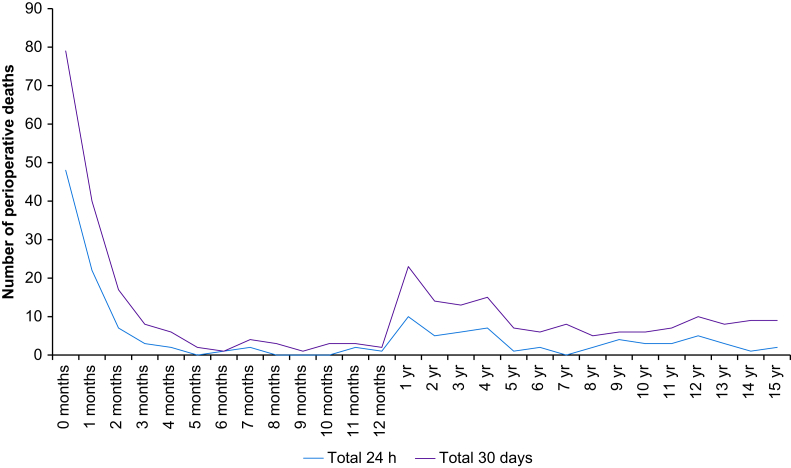
Age distribution of 24-h and 30-day perioperative deaths in the entire cohort. Age in months and years (yr).

The all-cause 24-h mortality was 142 (mortality rate 6.6:10,000 anaesthetics) and the all-cause 30-day mortality was 315 (14.7:10,000 anaesthetics) (Table 5). In the neonatal population, the all-cause 30-day mortality was 119 (286:10,000 anaesthetics). Children aged ≤3 yr at the time of operation accounted for most deaths, with patients under 3 months of age representing 9.1% of the population but 54% of the 24-h mortality and 43% of the 30-day mortality. The distribution of mortality according to age is displayed in Figure 2. Most neonatal deaths were registered at paediatric or university hospitals: 94% of the 24-h mortality and 93% of the 30-day mortality. More than one-third of deaths were in conjunction with cardiac surgery or cardiac catheterisation. Two of the 14 infants with intraoperative cardiac arrest in a paediatric hospital died within 24 h.

**Table 5:** Total 24-h and 30-day mortality count and incidence, grouped by hospital category. Count (range) of number of deaths in single hospitals and estimate of all-cause death per 10 000 procedures (95% confidence intervals).

Hospital category	24-h		30-day	
	Count (range)	*P* (95% CI)	Count (range)	*P* (95% CI)
Paediatric hospitals	113 (13–44)	15.1 (12.4–18.2)	257 (47–98)	34.3 (30.3–38.8)
University hospitals	20 (0–6)	4.8 (2.9–7.5)	36 (0–10)	8.7 (6.1–12.1)
County hospitals	8 (0–3)	1.2 (0.5–2.4)	20 (0–4)	3.1 (1.9–4.8)
District hospitals	1 (0–1)	0.3 (0.01–1.73)	2 (0–1)	0.62 (0.07–2.2)
Small hospitals	0 (0–0)	0 (0.0–22.1)	0 (0–0)	0 (0.0–22.1)

**Total**	**142 (0–44)**	**6.6 (0)**	**315 (0–98)**	**14.7 (0)**

In children with ASA physical status 1 and 2, there were zero and one death, respectively, within 24 h and one and 11 deaths within 30 days, respectively. The distribution of mortality according to ASA physical status is displayed in Supplementary Table S4. All 24-h and 30-day deaths in children with ASA physical status ≥3 were reported from paediatric (46 and 123), university (13 and 26), and county (2 and 11) hospitals (Table 5). As can be ascertained from the confidence intervals in Table 5, the estimated mortality, both 24-h and 30-day, was statistically significantly higher in the paediatric hospital group than in the other hospital categories, except for the 24-h mortality in small hospitals. Similarly, the estimated mortality was statistically significantly higher in non-paediatric university hospitals than in all other hospital categories, except paediatric hospitals.

## Discussion

In this large, nation-wide, register-based cohort study of all paediatric procedures involving anaesthesia services in Sweden during 2019–22, we found that 93% of the anaesthetics in children aged ≤12 months were performed in tertiary centres (paediatric or other university hospitals) and only 0.3% in district or smaller hospitals. The incidence of AEs and SAEs was 270:10,000 and 6.7:10,000, respectively. The mortality was low and the 24-h mortality almost exclusively (94%) confined to the tertiary centres.

The trend towards centralisation of paediatric anaesthesia is driven by the profession rather than by enforcement from regulating bodies. However, most of Sweden is sparsely populated, with large distances from rural and some regional areas to the specialised centres, and it is therefore not feasible to refer all otherwise healthy toddlers to paediatric hospitals. Thus, the health care system has to find the right balance between centralisation and maintaining the competence of general anaesthetists in rural hospitals that have to manage both minor procedures and emergencies locally. This is reflected by the fact that approximately one-third of children <4 yr of age were anaesthetised in county or smaller hospitals. In this context, it is reassuring that SAEs and deaths were more common at tertiary centres, in which we find most of the children with significant comorbidity.

The rate of intraoperative SAEs was lower than that in the APRICOT study; the rate of SAEs was 0.076% *vs* 5.3% severe critical events in APRICOT.^2^ The incidence of laryngospasm was 0.41% (or 0.07% as SAE) in this cohort compared with 1.3% in APRICOT. Similarly, the combined incidence of severe critical respiratory events was 5:10,000, which is one order of magnitude smaller than the 3% critical respiratory events in APRICOT. The incidence of aspiration was less than 1:10,000, which is at the lower end of previous audits (reported range 0.6–12 in 10,000).^13^ The lower number of AEs in the present study could reflect differences in practice but could also represent less stringent registration of aspiration events without sequelae (i.e. reporting bias). Moreover, the incidence of cardiac arrest in the present study (1:10,000) was similar to those in the Wake Up Safe and 7th National Audit Project (NAP7), which both reported incidences around 3:10,000.^14^, ^15^

AEs were less common in paediatric hospitals than in county hospitals. This could possibly support that surgery in this high-risk age group should be centralised to higher levels of care whenever possible.^16^^,^^17^ An opposing pattern is evident for mortality rate where county hospitals only accounted for 2% of registered 30-day mortality, whereas paediatric hospitals accounted for 89%. This would indicate that although children operated on in paediatric and university hospitals had a somewhat lower risk of complications, they also had a substantially higher risk of mortality. However, it should be noted that 87% of all operations on patients with ASA ≥3 were performed at paediatric or university hospitals. Thus, most of the mortality was registered at the hospitals performing the operations with highest risk on children with increased comorbidity.

Mortality in paediatric anaesthesia has declined in recent decades.^18^^,^^19^ The 30-day mortality has been reported to range from 10 to 41.6:10,000 anaesthetics in three large studies.^2^^,^^20^^,^^21^ In two of these, conducted in major tertiary centres in Australia and the Netherlands, the reported 24-h mortality was 13.4 and 13.1:10,000 anaesthetics, respectively.^20^^,^^21^ We report an overall mortality of 14.7 in 10,000 procedures, which is in line with the latter two studies but considerably higher than 0.01:10,000 anaesthesia-related mortality reported in the recent report from the Wake Up Safe register. In the present study, we could unfortunately not determine anaesthesia-related mortality.

In the neonatal subpopulation, the reported 30-day mortality ranges from 367 to 410:10,000 anaesthetics.^3^^,^^20^^,^^21^ In the present study, we found 286:10,000 anaesthetics.

### Strengths and limitations

This is the first study to provide detailed information on the distribution of paediatric anaesthesia in Sweden, and it is one of a handful of studies investigating the rate of AEs associated with paediatric anaesthesia in a single country. The study has several limitations. Although SPOR receives validated and complete datasets regarding automatically downloaded data, such as date and time of surgery and anaesthesia, type of anaesthesia, and airway management, the registration of AEs is more subjective, including reporting or not reporting when there is no AE, and grading of the severity of AEs that have occurred. It is possible that SAE data are less likely to be missing than less severe AE data, but compulsory reporting would probably lead to less missing data. Speculatively, for example, a laryngospasm that does not lead to sequelae will be registered by some but disregarded by others. Moreover, there is no consensus regarding thresholds for harmful hypotension or even desaturation.

In the present study, two hospitals did not report AEs, and data were missing in 12% of cases from the remaining 79 hospitals. This is a source of error when comparing rates of AEs with stringent audits with detailed case report forms such as the APRICOT, Wake Up Safe, and NAP7. We suggest that three measures are needed to increase the quality of AE data in SPOR: firstly, to develop a consensus regarding definitions of complications in paediatric anaesthesia; secondly, to implement a programme to increase reporting of AE, both quantitatively and qualitatively; and thirdly, when the first two measures are in place, to evaluate and validate the quality of AE reporting in SPOR.

The exact time of death was not available in the dataset from SPOR, leading to mortality being calculated on the basis of the available dates. Thus 24-h mortality included all deaths on the same date and the date after the last registered procedure, and 30-day mortality consisted of all deaths recorded up to 31 days after the last operation. This could lead to a slight overestimation of the ‘true’ 24-h mortality in the SPOR cohort. Similarly, only birth year and month were provided in the data, leading to age at operation in months for patients under 1 yr of age being based on the birth and operating month. Although the overall mortality data in the present large cohort are reliable, as SPOR is continuously updated from the Swedish Cause of Death Register, it does not reflect mortality directly caused by anaesthesia. The number of deaths directly caused by inadequate anaesthesia management or associated with anaesthesia in Sweden is likely to be significantly lower than the overall mortality reported in the present study. Furthermore, although Sweden has a tax-funded health care system, a few small private clinics perform office-based procedures in children without reporting their data to SPOR and could thus not be included in the present study.

Finally, Sweden is an affluent, unevenly populated country with a developed government-run free-for-all health care system. Although results may not be generalised to low- and middle-income settings, similar conditions exist in many Northern European countries.

We conclude that although most operations in children (54%) were performed in paediatric and university hospitals, county hospitals handle a large portion of the caseload of ages ≥3 yr. Almost all registered deaths occurred in patients under the age of 3 yr at paediatric hospitals, where most operations on patients with significant comorbidity were performed. Reporting of AEs in SPOR needs to be improved, and a process to achieve international consensus regarding definitions and principles of reporting is warranted. Future studies will benefit from standardised reporting of AEs.

## Authors’ contributions

Study conception and design: PF

Data collection: BBE

Data analysis: BBE, ARM, PF

Writing the first draft of the manuscript: BBE

Writing the manuscript: PF

Critical revision of the manuscript: KS, RF, PF

All authors approved the final version of the manuscript.

## Acknowledgements

We thank the staff at Uppsala Clinical Research Centre for providing SPOR data and Johan Bring at Statisticon AB for expert statistical advice.

## Declaration of interest

The authors declare no conflicts of interest.

## Funding

Departmental funding (Uppsala University).
