# Supplementary material for: Distribution and outcomes of paediatric anaesthesia services in Sweden: an epidemiological study
Source: Br J Anaesth. 2024 Aug 1;133(4):804–9. doi: 10.1016/j.bja.2024.07.007 (PMC11443129; doi:10.1016/j.bja.2024.07.007)
Supplement: Multimedia component 1 [file mmc1.docx]

**Supplemental Figure S1**. Flowchart. Cases without adverse events (AEs) entries were excluded from the analysis of AEs but included in the analysis of distribution and mortality.

**Initial cohort**

Number of hospitals = 81

Number of cases = 214,964

**Analysis of distribution and mortality**

Number of hospitals = 81

Number of cases = 214,964

**Analysis of AEs**

Number of hospitals = 79

Number of cases = 174,316

*Cases excluded because the hospital PDMS did not transfer AEs to SPOR*

Number of hospitals = 2

Number of cases = 17,034

*Cases excluded due to incomplete adverse events registration:*

Number of cases = 23,614
