# Supplementary material for: Distribution and outcomes of paediatric anaesthesia services in Sweden: an epidemiological study
Source: Br J Anaesth. 2024 Aug 1;133(4):804–9. doi: 10.1016/j.bja.2024.07.007 (PMC11443129; doi:10.1016/j.bja.2024.07.007)
Supplement: Multimedia component 2 [file mmc2.docx]

**Supplemental Table S1.** Distribution of operations, grouped by age at operation and hospital category. Count (percentage of row).

| **Age at operation** | **Paediatric hospitals** | **University hospitals** | **County hospitals** | **District hospitals** | **Smaller units** | **Total** |
| --- | --- | --- | --- | --- | --- | --- |
| 0 | 11656 (72%) | 3439 (21%) | 1067 (7%) | 48 (0%) | 1 (0%) | 16211 |
| 1 | 6838 (47%) | 3099 (21%) | 3528 (24%) | 936 (6%) | 230 (2%) | 14631 |
| 2 | 5796 (38%) | 2860 (19%) | 4870 (32%) | 1687 (11%) | 231 (1%) | 15444 |
| 3 | 5346 (33%) | 2766 (17%) | 5447 (33%) | 2661 (16%) | 187 (1%) | 16407 |
| 4 | 5042 (31%) | 2675 (17%) | 5339 (33%) | 2946 (18%) | 109 (1%) | 16111 |
| 5 | 4924 (31%) | 2644 (17%) | 5183 (33%) | 2886 (18%) | 137 (1%) | 15774 |
| 6 | 4304 (30%) | 2417 (17%) | 4576 (32%) | 2706 (19%) | 152 (1%) | 14155 |
| 7 | 4077 (31%) | 2307 (18%) | 4103 (32%) | 2426 (19%) | 87 (1%) | 13000 |
| 8 | 3484 (30%) | 2196 (19%) | 3845 (33%) | 2081 (18%) | 120 (1%) | 11726 |
| 9 | 3498 (31%) | 2187 (19%) | 3636 (32%) | 1907 (17%) | 61 (1%) | 11289 |
| 10 | 3276 (29%) | 2227 (20%) | 3846 (34%) | 1876 (17%) | 66 (1%) | 11291 |
| 11 | 3260 (29%) | 2208 (19%) | 3890 (34%) | 1950 (17%) | 68 (1%) | 11376 |
| 12 | 3414 (30%) | 2291 (20%) | 3803 (33%) | 1865 (16%) | 43 (0%) | 11416 |
| 13 | 3451 (30%) | 2426 (21%) | 3637 (32%) | 1900 (17%) | 59 (1%) | 11473 |
| 14 | 3540 (29%) | 2705 (22%) | 3843 (32%) | 2002 (16%) | 58 (0%) | 12148 |
| 15 | 2917 (23%) | 2822 (23%) | 4316 (34%) | 2397 (19%) | 60 (0%) | 12512 |
| **Total** | **74823** | **41269** | **64929** | **32274** | **1669** | **214964** |
