# Supplementary material for: Distribution and outcomes of paediatric anaesthesia services in Sweden: an epidemiological study
Source: Br J Anaesth. 2024 Aug 1;133(4):804–9. doi: 10.1016/j.bja.2024.07.007 (PMC11443129; doi:10.1016/j.bja.2024.07.007)
Supplement: Multimedia component 3 [file mmc3.docx]

**Supplemental Table S2.** Distribution of ASA-class across age groups and hospital categories.

|  | **Paediatric hospitals** | | **University hospitals** | | **County hospitals** | | **District hospitals** | | **Smaller units** | | **Total Count** |
| --- | --- | --- | --- | --- | --- | --- | --- | --- | --- | --- | --- |
|  | **Count** | **% of row** | **Count** | **% of row** | **Count** | **% of row** | **Count** | **% of row** | **Count** | **% of row** |  |
| **Neonates** | **3494** | **83,93%** | **553** | **13,28%** | **111** | **2,67%** | **5** | **0,12%** | **0** | **0,00%** | **4163** |
| ASA 1 | 246 | 71,51% | 71 | 20,64% | 27 | 7,85% | 0 | 0,00% | 0 | 0,00% | 344 |
| ASA 2 | 555 | 77,19% | 143 | 19,89% | 20 | 2,78% | 1 | 0,14% | 0 | 0,00% | 719 |
| ASA 3 | 1073 | 85,91% | 166 | 13,29% | 10 | 0,80% | 0 | 0,00% | 0 | 0,00% | 1249 |
| ASA 4 | 401 | 81,67% | 88 | 17,92% | 2 | 0,41% | 0 | 0,00% | 0 | 0,00% | 491 |
| ASA 5 | 21 | 72,41% | 6 | 20,69% | 2 | 6,90% | 0 | 0,00% | 0 | 0,00% | 29 |
| Not reported | 1196 | 90,13% | 78 | 5,88% | 49 | 3,69% | 4 | 0,30% | 0 | 0,00% | 1327 |
| Unknown | 2 | 50,00% | 1 | 25,00% | 1 | 25,00% | 0 | 0,00% | 0 | 0,00% | 4 |
| **Infants** | **8162** | **67,75%** | **2886** | **23,95%** | **956** | **7,93%** | **43** | **0,36%** | **1** | **0,01%** | **12048** |
| ASA 1 | 1961 | 51,77% | 1321 | 34,87% | 497 | 13,12% | 9 | 0,24% | 0 | 0,00% | 3788 |
| ASA 2 | 2207 | 68,75% | 722 | 22,49% | 273 | 8,50% | 8 | 0,25% | 0 | 0,00% | 3210 |
| ASA 3 | 2496 | 79,59% | 575 | 18,34% | 63 | 2,01% | 1 | 0,03% | 1 | 0,03% | 3136 |
| ASA 4 | 291 | 77,19% | 84 | 22,28% | 2 | 0,53% | 0 | 0,00% | 0 | 0,00% | 377 |
| ASA 5 | 8 | 72,73% | 3 | 27,27% | 0 | 0,00% | 0 | 0,00% | 0 | 0,00% | 11 |
| Not reported | 1189 | 79,11% | 176 | 11,71% | 113 | 7,52% | 25 | 1,66% | 0 | 0,00% | 1503 |
| Unknown | 10 | 43,48% | 5 | 21,74% | 8 | 34,78% | 0 | 0,00% | 0 | 0,00% | 23 |
| **Toddlers** | **6838** | **46,74%** | **3099** | **21,18%** | **3528** | **24,11%** | **936** | **6,40%** | **230** | **1,57%** | **14631** |
| ASA 1 | 2595 | 36,53% | 1493 | 21,02% | 2280 | 32,09% | 617 | 8,69% | 119 | 1,68% | 7104 |
| ASA 2 | 1818 | 46,78% | 932 | 23,98% | 841 | 21,64% | 191 | 4,92% | 104 | 2,68% | 3886 |
| ASA 3 | 1481 | 70,12% | 485 | 22,96% | 134 | 6,34% | 10 | 0,47% | 2 | 0,09% | 2112 |
| ASA 4 | 79 | 62,70% | 41 | 32,54% | 6 | 4,76% | 0 | 0,00% | 0 | 0,00% | 126 |
| ASA 5 | 1 | 25,00% | 3 | 75,00% | 0 | 0,00% | 0 | 0,00% | 0 | 0,00% | 4 |
| Not reported | 843 | 62,72% | 144 | 10,71% | 235 | 17,49% | 117 | 8,71% | 5 | 0,37% | 1344 |
| Unknown | 21 | 38,18% | 1 | 1,82% | 32 | 58,18% | 1 | 1,82% | 0 | 0,00% | 55 |
| **Early childhood** | **16184** | **33,74%** | **8301** | **17,31%** | **15656** | **32,64%** | **7294** | **15,21%** | **527** | **1,10%** | **47962** |
| ASA 1 | 4584 | 19,19% | 3764 | 15,76% | 10205 | 42,72% | 5027 | 21,04% | 310 | 1,30% | 23890 |
| ASA 2 | 4231 | 33,29% | 2556 | 20,11% | 4133 | 32,52% | 1595 | 12,55% | 193 | 1,52% | 12708 |
| ASA 3 | 4344 | 66,85% | 1297 | 19,96% | 732 | 11,27% | 109 | 1,68% | 16 | 0,25% | 6498 |
| ASA 4 | 160 | 61,07% | 86 | 32,82% | 16 | 6,11% | 0 | 0,00% | 0 | 0,00% | 262 |
| ASA 5 | 5 | 62,50% | 1 | 12,50% | 1 | 12,50% | 1 | 12,50% | 0 | 0,00% | 8 |
| ASA 6 | 0 | 0,00% | 1 | 50,00% | 0 | 0,00% | 1 | 50,00% | 0 | 0,00% | 2 |
| Not reported | 2847 | 63,18% | 588 | 13,05% | 516 | 11,45% | 547 | 12,14% | 8 | 0,18% | 4506 |
| Unknown | 13 | 14,77% | 8 | 9,09% | 53 | 60,23% | 14 | 15,91% | 0 | 0,00% | 88 |
| **School children** | **40145** | **29,48%** | **26430** | **19,41%** | **44678** | **32,81%** | **23996** | **17,62%** | **911** | **0,67%** | **136160** |
| ASA 1 | 15145 | 19,96% | 13469 | 17,75% | 29853 | 39,34% | 16848 | 22,20% | 561 | 0,74% | 75876 |
| ASA 2 | 11344 | 32,35% | 7592 | 21,65% | 10972 | 31,29% | 4847 | 13,82% | 307 | 0,88% | 35062 |
| ASA 3 | 7785 | 56,41% | 3510 | 25,43% | 2111 | 15,30% | 365 | 2,64% | 29 | 0,21% | 13800 |
| ASA 4 | 286 | 55,53% | 181 | 35,15% | 44 | 8,54% | 4 | 0,78% | 0 | 0,00% | 515 |
| ASA 5 | 6 | 60,00% | 4 | 40,00% | 0 | 0,00% | 0 | 0,00% | 0 | 0,00% | 10 |
| ASA 6 | 1 | 33,33% | 2 | 66,67% | 0 | 0,00% | 0 | 0,00% | 0 | 0,00% | 3 |
| Not reported | 5542 | 51,73% | 1659 | 15,49% | 1603 | 14,96% | 1895 | 17,69% | 14 | 0,13% | 10713 |
| Unknown | 36 | 19,89% | 13 | 7,18% | 95 | 52,49% | 37 | 20,44% | 0 | 0,00% | 181 |
| **Grand Total** | **74823** | **34,81%** | **41269** | **19,20%** | **64929** | **30,20%** | **32274** | **15,01%** | **1669** | **0,78%** | **214964** |
