# Supplementary material for: Distribution and outcomes of paediatric anaesthesia services in Sweden: an epidemiological study
Source: Br J Anaesth. 2024 Aug 1;133(4):804–9. doi: 10.1016/j.bja.2024.07.007 (PMC11443129; doi:10.1016/j.bja.2024.07.007)
Supplement: Multimedia component 4 [file mmc4.docx]

**Supplemental Table S3.** Number of adverse events registered at the 5 different hospital categories. Grade 0 - designate absence of AEs. True adverse events range from Grade 1 (no effect on postoperative care), Grade 2 (affects provision of care in the PACU, but not further postoperative care), Grade 3 (affects the provision of care in the postoperative unit with prolonged care and/or extra observation), Grade 4 (affects the provision of care so that postoperative intensive care is required), to Grade 5 (entails probable lasting morbidity or mortality).

|  |  |  |  |  |  |  |
| --- | --- | --- | --- | --- | --- | --- |
| **Row Labels** | **Paediatric hospitals** | **University hospitals** | **County hospitals** | **District hospitals** | **Smaller units** | **Total** |
| Grade 0 | 44,006 | 35,671 | 57,982 | 29,786 | 1,513 | 168,958 |
| Grade 1 | 824 | 1036 | 1553 | 558 | 15 | 3986 |
| Grade 2 | 229 | 209 | 411 | 144 | 6 | 999 |
| Grade 3 | 66 | 60 | 89 | 25 | 0 | 240 |
| Grade 4 | 39 | 24 | 47 | 12 | 0 | 122 |
| Grade 5 | 5 | 2 | 4 | 0 | 0 | 11 |
| Not reported | 12652 | 4267 | 4843 | 1717 | 135 | 23,614 |
| **Total** | **57,821** | **41,269** | **64,929** | **32,242** | **1669** | **197,930** |
