# Supplementary material for: Distribution and outcomes of paediatric anaesthesia services in Sweden: an epidemiological study
Source: Br J Anaesth. 2024 Aug 1;133(4):804–9. doi: 10.1016/j.bja.2024.07.007 (PMC11443129; doi:10.1016/j.bja.2024.07.007)
Supplement: Multimedia component 5 [file mmc5.docx]

**Supplemental Table S4**. Mortality grouped by ASA class.

|  | **Paediatric hospitals** | | **University hospitals** | | **County hospitals** | | **District hospitals** | | **Smaller units** | | **Total** | |
| --- | --- | --- | --- | --- | --- | --- | --- | --- | --- | --- | --- | --- |
| **ASA class** | **24h** | **30d** | **24h** | **30d** | **24h** | **30d** | **24h** | **30d** | **24h** | **30d** | **24h** | **30d** |
| ASA I | 0 | 0 | 0 | 1 | 0 | 0 | 0 | 0 | 0 | 0 | 0 | 1 |
| ASA II | 0 | 7 | 0 | 1 | 1 | 3 | 0 | 0 | 0 | 0 | 1 | 11 |
| ASA III | 9 | 54 | 1 | 9 | 1 | 9 | 0 | 0 | 0 | 0 | 11 | 72 |
| ASA IV | 26 | 57 | 4 | 9 | 0 | 1 | 0 | 0 | 0 | 0 | 30 | 67 |
| ASA V | 10 | 11 | 5 | 5 | 1 | 1 | 0 | 1 | 0 | 0 | 16 | 18 |
| ASA VI | 1 | 1 | 3 | 3 | 0 | 0 | 1 | 1 | 0 | 0 | 5 | 5 |
| Not reported | 67 | 127 | 7 | 8 | 5 | 6 | 0 | 0 | 0 | 0 | 79 | 141 |
| **Grand Total** | **113** | **257** | **20** | **36** | **8** | **20** | **1** | **2** | **0** | **0** | **142** | **315** |
